# Effect of *Ruellia tuberosa* L. on aorta endothelial damage‐associated factors in high‐fat diet and streptozotocin‐induced type 2 diabetic rats

**DOI:** 10.1002/fsn3.1233

**Published:** 2019-10-09

**Authors:** Chih‐Yuan Ko, Ru‐Hai Lin, Yangming Martin Lo, Wen‐Chang Chang, Da‐Wei Huang, James Swi‐Bea Wu, Yu‐Fang Chang, Wen‐Chung Huang, Szu‐Chuan Shen

**Affiliations:** ^1^ Department of Respiratory and Critical Care Medicine The Second Affiliated Hospital of Fujian Medical University Quanzhou China; ^2^ Department of Clinical Nutrition The Second Affiliated Hospital of Fujian Medical University Quanzhou China; ^3^ Respiratory Medicine Center of Fujian Province Quanzhou China; ^4^ The Sleep Medicine Key Laboratory of Fujian Province Universities Quanzhou China; ^5^ Department of Endocrinology and Metabolism The Second Affiliated Hospital of Fujian Medical University Quanzhou China; ^6^ Institute for Advanced Study Shenzhen University Shenzhen China; ^7^ Department of Food Science National Chiayi University Chiayi City Taiwan; ^8^ Department of Biotechnology and Food Technology Southern Taiwan University of Science and Technology Tainan City Taiwan; ^9^ Graduate Institute of Food Science and Technology National Taiwan University Taipei Taiwan; ^10^ Graduate Program of Nutrition Science National Taiwan Normal University Taipei Taiwan; ^11^ Graduate Institute of Health Industry Technology Chang Gung University of Science and Technology Taoyuan Taiwan

**Keywords:** antioxidative enzyme, aorta dysfunction, endothelial damage‐associated factors, *Ruellia tuberosa* L., type 2 diabetes mellitus

## Abstract

Hyperglycemia plays crucial roles in vascular disease development, including macrovascular and microvascular diseases from diabetes mellitus (DM). Our previous study demonstrated that *Ruellia tuberosa* L. (RTL) aqueous and ethanol extracts alleviate hyperglycemia and inhibit insulin resistance in diabetic rats. This study investigated the protective effect of RTL ethanol extract against aorta dysfunction in high‐fat diet (HFD) and streptozotocin (STZ)‐induced type 2 DM (T2DM) rats. Results showed that RTL ethanol extract (100 and 400 mg/kg BW/day) ameliorated serum lipid profiles, including triglyceride, free fatty acid, low‐density lipoprotein cholesterol, very low‐density lipoprotein cholesterol, and high‐density lipoprotein cholesterol levels. It also significantly reduced the level of serum cytokines such as tumor necrosis factor‐α (TNF‐α) and interleukin‐6 in T2DM rats. Additionally, RTL extract decreased endothelin‐1 and endothelial nitric oxide contents, reduced the level of cell adhesion factors, including monocyte chemoattractant protein‐1 and cell adhesion factor vascular cell adhesion molecule‐1, while decreasing content of damage factors, namely tissue factor and von Willebrand factor in aortic tissues of diabetic rats. Equally noteworthy is that RTL extract enhanced the activity of aorta antioxidative enzymes, including superoxidase dismutase and catalase in diabetic rats, suggesting that RTL ethanol extract may ameliorate aorta dysfunction via enhancing aortic antioxidative enzyme activity, which subsequently suppresses aorta endothelial damage‐associated factors in HFD with STZ‐induced T2DM rats.

## 1 INTRODUCTION

Diabetes mellitus (DM) is a chronic metabolic disease commonly known to result in carbohydrate, protein, and lipid metabolism disorders in patients, of whom 95% have type 2 DM (T2DM; ADA, 2015). Insulin resistance (IR) is the hallmark characteristic of T2DM. The pathogenesis of IR is mainly attributed to reduced ability to process glucose as well as decline in insulin sensitivity in peripheral tissue, ultimately leading to hyperinsulinemia and hyperglycemia (King, Park, & Li, 2016). These conditions easily lead to increased oxidative stress as evidenced by chronic inflammatory responses, consequently resulting in several diabetic complications involving macrovascular and microvascular diseases (Al Disi, Anwar, & Eid, 2016; King et al., 2016).

Evidence has shown that vascular endothelial cell dysfunction of the large blood vessels (macrovascular system), including the arteries and aorta, and the capillaries/microvessels (microvascular system) are positively associated with the onset of cardiovascular complications associated with diabetes (Lüscher, Creager, Beckman, & Cosentino, 2003). As one of the most frequently occurring and most observed macrovascular complications from DM, atherosclerosis can lead to premature coronary artery disease, increased risk of cerebrovascular disease, and increased occurrence rate of peripheral vascular disease (Lüscher et al., 2003).

Several risk factors are recognized to be involved in the progression of diabetic vascular complications, including IR or insulin deficiency, hyperglycemia, and dyslipidemia, in which inflammatory responses mediate their pathogenesis (King et al., 2016; Zheng et al., 2013). Zheng et al. (2013) demonstrated that cascades of nuclear factor kappa B (NF‐κB) activate inflammatory factors such as cytokines (e.g., interleukin‐1β [IL‐1β], IL‐6, and tumor necrosis factor‐α [TNF‐α]) and adhesion molecules (e.g., monocyte chemoattractant protein‐1 [MCP‐1] and intracellular cell adhesion molecule‐1 [ICAM‐1]). These factors were observed to be elevated in in vitro high glucose cultured human endothelial cell lines and in vivo in the high‐fat diet (HFD) plus streptozotocin (STZ)‐induced diabetic rats. Long‐term hyperglycemia also disrupts the balance between nitric oxide (NO) and reactive oxygen species (ROS), subsequently resulting in endothelial cell dysfunction (Creager, Luscher, Cosentino, & Beckman, 2003). In addition, lipid accumulation is attributed to the occurrences of IR and hyperglycemia and causes inflammatory responses by activating the NF‐κB transmission pathway (Cavelti‐Weder et al., 2012), subsequently elevating thrombosis and the expression of several factors that mediate vascular endothelial cell damage such as endothelin‐1 (ET‐1), tissue factor (TF), and von Willebrand factor (vWF; Frankel et al., 2008; Holy & Tanner, 2010; Paneni, Beckman, Creager, & Cosentino, 2013).

*Ruellia tuberosa* L. (RTL) has been used as folk medicine in East Asia for treating diabetes for a couple decades and contains a wide range of pharmacological benefits such as antioxidant, anti‐inflammatory, and anticancer properties (Chothani, Patel, Mishra, & Vaghasiya, 2010; Rajan, Kishor Kumar, Satheesh Kumar, Swathi, & Haritha, 2012). The phytochemicals in RTL have been characterized to encompass three major constituents (i.e., flavonoids, triterpenes, and sterols) besides alkaloids, lignans, betulin, flavone glycoside, syringic acid, coumaric acid, vanillic acid, apigenin, apigenin rutinoside, apigenin glucoside, apigenin‐7‐O‐glucuronide, luteolin, luteolin glucoside, cirsimaritin, cirsimarin, pedalitin, cirsiliol 4‐glucoside, sorbifolin, 3,5‐diglucoside, and indole‐3‐carboxaldehyde (Wulan, Utomo, & Mahdi, 2015). The ethanol leaf extracts of RTL have been reported to improve dyslipidemia in alloxan‐induced type 1 diabetic rats (Ananthakrishnan & Doss, 2010, 2013). We recently demonstrated that aqueous and ethanol extracts from RTL ameliorate blood glucose homeostasis by alleviating IR in skeletal muscles (Ko et al., 2018) and reduce hepatic detoxification function by increasing hepatic antioxidative enzyme activities in HFD and STZ‐induced diabetic rats (Chang et al., 2018). However, there remains a void in the literature regarding how RTL interact with aortic systems in diabetic patients, especially in the T2DM paradigm. The present study aims to investigate the protective potential of RTL against aorta dysfunction and its possible mechanism in a T2DM rat model.

## 2 MATERIALS AND METHODS

### 2.1 Preparation of RTL extracts

*Ruellia tuberosa* L. was gathered from the Herb Light farm, Yi‐Lan County, Taiwan, in May 2014. The RTL stems and leaves were washed, drained, weighed, sliced, and freeze‐dried. Each 1 g of dried material was extracted with 6 ml 95% ethanol (1:6, w/v) at 4°C for 72 hr and filtered through cheese cloth. The filtrate was filtered twice through Whatman No. 1 filter paper and, then, centrifuged at 4,700 *g* for 20 min. The supernatant was vacuum concentrated using a rotary evaporator at <40°C. The concentrate was freeze‐dried and kept at 80°C until use. The freeze‐dried extract powder appeared brownish green.

### 2.2 Animal experimental procedures

Forty‐two four‐week‐old male Wistar rats were procured from the National Laboratory Animal Center, Taipei, Taiwan. The room conditions and treatment procedures were in accordance with the National Institutes of Health Guide for the Care and Use of Laboratory Animals, and all protocols were approved by the Institutional Animal Care and Use Committee of National Taiwan Normal University, Taipei, Taiwan (approval no. 103042). The rats were housed in a temperature‐ (22 ± 1°C) and humidity‐controlled (50 ± 20%) room under a 12‐hr light/dark cycle (lights on from 08:00 to 20:00) with free access to food and water. After allowing the rats to adapt to their environment for 1 week, they were fed an HFD (60% calories from fat) for 4 weeks. During the 5th and 6th weeks, STZ (28 and 15 mg/kg body weight, respectively, dissolved in 0.1 M sodium citrate buffer at pH 4.5) was intraperitoneally injected into each HFD rat to induce diabetes. After STZ injection, the rats were supplied with drinking water containing 5% sucrose for 48 hr to reduce early death that might be resulted from insulin discharge from partially injured pancreatic islets. Seventy‐two hours later, the rats were tested for blood sugar. The diabetic rats were then fed HFD for 6 weeks prior to experimental procedures to guarantee the stable phenomena of hyperglycemia. For the experimental design, the rats were divided into five groups of six rats each: normal group, rats fed with normal diet; DM group, diabetic rats fed with HFD (60% calories from fat) as the negative control; DM+Pio group, diabetic rats fed with HFD and orally administered pioglitazone (Pio; 30 mg/kg body weight) daily for 4 weeks as the positive control; and DM+E100 and DM+E400 groups, diabetic rats fed with HFD and orally administered RTL ethanolic extracts (100 or 400 mg/kg body weight, respectively) daily for 4 weeks, respectively. All the rats were sacrificed at the end of the experiment.

### 2.3 Blood and tissue samples

After the rats were sacrificed, blood samples were collected and allowed to clot for 30 min at room temperature and centrifuged twice at 3,000 *g* for 20 min each to obtain the serum. The aortic tissue was separated and removed from the heart immediately after death and stored at −80°C until use.

### 2.4 Serum lipid profiles, cytokines, and cell adhesion factors

Enzyme‐linked immunosorbent assay (ELISA) kits to assess levels of free fatty acid (FFA), total cholesterol, triglyceride, high‐density lipoprotein cholesterol (HDL‐C), low‐density lipoprotein cholesterol (LDL‐C), very low‐density lipoprotein cholesterol (VLDL‐C), IL‐6, and TNF‐α in the rats were purchased from Randox Laboratories (Crumlin Co.). Vascular cell adhesion molecule‐1 (VCAM‐1) and ICAM‐1 were purchased from SunLong Biotech. Biochemical analyses were conducted according to each supplier's protocols.

### 2.5 Aortic tissue protein preparation and concentration

Bovine serum albumin was used as the standard protein and diluted to a final concentration range of 0–500 μg/ml. The aortic tissue proteins were diluted approximately 100‐fold to bring its concentration within the standard range. The protein concentration was measured at 595 nm using the Bradford method with a Bio‐Rad protein assay kit and a spectrophotometer.

### 2.6 Aortic antioxidative enzyme activity

Superoxidase dismutase (SOD) and catalase activities were measured as described previously (Chang et al., 2018).

### 2.7 Aortic cell adhesion factor

Kits for assessing aortic tissue MCP‐1 (Abcam) and VCAM‐1 (SunLong Biotech) were used according to each supplier's protocols.

### 2.8 Aortic eNOS, ET‐1 and damage factor

Enzyme‐linked immunosorbent assay kits to assess aortic tissue endothelial nitric oxide (eNOS), ET‐1, TF, and vWF were purchased from SunLong Biotech and were used according to the manufacturer's protocols.

### 2.9 Statistical analyses

Values are presented as the mean ± standard deviation (*SD*) using SAS v. 9.4 (SAS Institute Inc) and one‐way analysis of variance and Duncan's new multiple range tests. *p* < .05 was considered statistically significant.

## 3 RESULTS

### 3.1 Effect of RTL on lipid profiles in HFD and STZ‐induced T2DM diabetic rats

Table 1 shows that serum TG and VLDL‐C contents in the DM group were significantly higher than those in the normal group (*p* < .05). Serum TG and VLDL‐C contents in DM+E100 and DM+E400 groups were significantly reduced compared with DM group. Furthermore, serum FFA and LDL‐C levels in DM+E400 group were lower than those in the DM group (*p* < .05) by 18.07% and 33.33%, respectively. Moreover, HDL‐C level in DM+E400 group was higher than that in the DM group (*p* < .05) by 21.40%.

**Table 1:** Profiles of serum biochemical lipid in high‐fat diet and streptozotocin‐induced type 2 diabetic rats after treating with *Ruellia tuberosa* L. (RTL) extracts for 4 weeks

	Normal	DM	DM+Pio	DM+E100	DM+E400
Triglyceride (mg/dl)	78.0 ± 7.2^de^	145.5 ± 26.0^a^	60.7 ± 11.1^e^	103.7 ± 23.8^bc^	125.0 ± 16.6^ab^
Free fatty acid (mmol/L)	1.11 ± 0.22^a^	1.09 ± 0.14^a^	0.74 ± 0.08^c^	1.06 ± 0.22^ab^	0.89 ± 0.11^bc^
Total cholesterol (mg/dl)	63.3 ± 2.3^b^	67.2 ± 7.8^ab^	72.2 ± 3.7^a^	66.3 ± 7.2^ab^	63.7 ± 5.1^b^
High‐density lipoprotein cholesterol (mg/dl)	53.8 ± 5.6^ab^	47.5 ± 5.1^b^	54.7 ± 4.5^a^	58.0 ± 2.1^a^	57.7 ± 4.5^a^
Low‐density lipoprotein cholesterol (mg/dl)	9.33 ± 0.52^ab^	10.00 ± 4.82^a^	12.50 ± 1.87^a^	8.17 ± 1.60^b^	6.67 ± 1.21^b^
Very low‐density lipoprotein cholesterol (mg/dl)	3.50 ± 0.84^b^	5.17 ± 0.75^a^	2.83 ± 0.98^b^	3.17 ± 0.98^b^	1.33 ± 0.52^c^

Note

Normal: Normal diet; DM: high‐fat diet (HFD; 60% fat) plus STZ (28 mg/kg body weight, i.p.) induced diabetic rats; DM+Pio: DM rats gavaged with pioglitazone (30 mg/kg body weight) for 4 weeks; DM+E100: DM rats gavaged with RTL ethanol extract (100 mg/kg body weight) for 4 weeks; DM+E400: DM rats gavaged with RTL ethanol extract (400 mg/kg body weight) for 4 weeks. Values were calculated as the mean ± *SD*, *n* = 6 for each group. a–c letters = significant differences among all samples tested (*p* < .05).

### 3.2 Effect of RTL on eNOS and ET‐1 in aortic tissues of HFD and STZ‐induced diabetic rats

**Figure 1:**
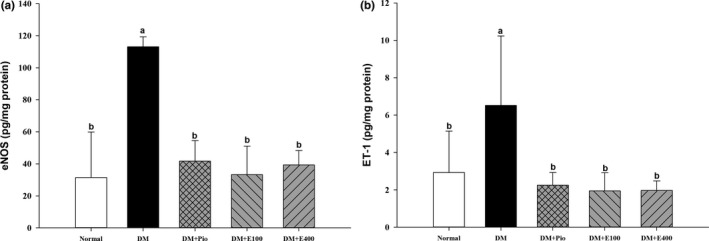
Endothelial nitric oxide synthase (eNOS; a) and endothelin‐1 (ET‐1; b) in aortas of high‐fat diet and streptozotocin‐induced type 2 diabetic rats treated with *Ruellia tuberosa* L. (RTL) extracts for 4 weeks. Normal: Normal diet; DM: high‐fat diet (HFD; 60% fat) plus STZ (28 mg/kg body weight, i.p.) induced diabetic rats; DM+Pio: DM rats gavaged with pioglitazone (30 mg/kg body weight) for 4 weeks; DM+E100: DM rats gavaged with RTL ethanol extract (100 mg/kg body weight) for 4 weeks; DM+E400: DM rats gavaged with RTL ethanol extract (400 mg/kg body weight) for 4 weeks. Values were calculated as the mean ± *SD*, *n* = 6 for each group. a–b letters = significant differences among all samples tested (*p* < .05)

The aorta eNOS and ET‐1 levels in the DM group were higher than those in the normal group and were significantly decreased in the DM+Pio, DM+E100, and DM+E400 groups (*p* < .05; Figure 1).

### 3.3 Effect of RTL on cell adhesion factors in aortic tissues of HFD and STZ‐induced diabetic rats

**Figure 2:**
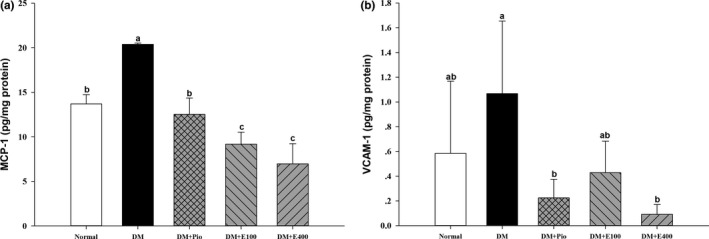
Monocyte chemoattractant protein‐1 (MCP‐1; a) and vascular cell adhesion molecule‐1 (VCAM‐1; b) levels in aortas of high‐fat diet and streptozotocin‐induced type 2 diabetic rats treated with *Ruellia tuberosa* L. (RTL) extracts for 4 weeks. Normal: Normal diet; DM: high‐fat diet (HFD; 60% fat) plus STZ (28 mg/kg body weight, i.p.) induced diabetic rats; DM+Pio: DM rats gavaged with pioglitazone (30 mg/kg body weight) for 4 weeks; DM+E100: DM rats gavaged with RTL ethanol extract (100 mg/kg body weight) for 4 weeks; DM+E400: DM rats gavaged with RTL ethanol extract (400 mg/kg body weight) for 4 weeks. Values were calculated as the mean ± *SD*, *n* = 6 for each group. a–c letters = significant differences among all samples tested (*p* < .05)

Figure 2 shows that MCP‐1 (20.40 ± 0.13 ng/mg protein) and VCAM‐1 (1.07 ± 0.59 ng/mg protein) levels in the aortic tissues of DM group were significantly higher than those in the normal group (*p* < .05). The aorta MCP‐1 and CAM‐1 levels in the DM+E400 group were decreased (*p* < .05) by 65.78% and 91.46%, respectively, compared with the DM group.

### 3.4 Effect of RTL on serum cytokines in HFD and STZ‐induced diabetic rats

Table 2 shows that serum IL‐6 and TNF‐α levels were significantly higher in the DM group than in the normal group (*p* < .05). The serum IL‐6 and TNF‐α levels in the DM+E400 group were lower than that in the DM group (*p* < .05) by 50.94% and 38.80%, respectively.

**Table 2:** Serum cytokines in high‐fat diet and streptozotocin‐induced type 2 diabetic rats after treating with *Ruellia tuberosa* L. (RTL) extracts for 4 weeks

	Normal	DM	DM+Pio	DM+E100	DM+E400
Tumor necrosis factor‐α (pg/ml)	51.5 ± 3.0^b^	90.3 ± 7.8^a^	45.7 ± 6.8^b^	53.1 ± 7.1^b^	55.2 ± 7.8^b^
Interleukin‐6 (pg/ml)	170.3 ± 60.7^ab^	190.9 ± 30.5^a^	153.4 ± 42.3^abc^	127.2 ± 25.4^bcd^	93.6 ± 28.8^d^

Note

Normal: Normal diet; DM: high‐fat diet (HFD; 60% fat) plus STZ (28 mg/kg body weight, i.p.) induced diabetic rats; DM+Pio: DM rats gavaged with pioglitazone (30 mg/kg body weight) for 4 weeks; DM+E100: DM rats gavaged with RTL ethanol extract (100 mg/kg body weight) for 4 weeks; DM+E400: DM rats gavaged with RTL ethanol extract (400 mg/kg body weight) for 4 weeks. Values were calculated as the mean ± *SD*, *n* = 6 for each group. a–c letters = significant differences among all samples tested (*p* < .05).

### 3.5 Effect of RTL on damage factors in aortic tissues of HFD and STZ‐induced diabetic rats

**Figure 3:**
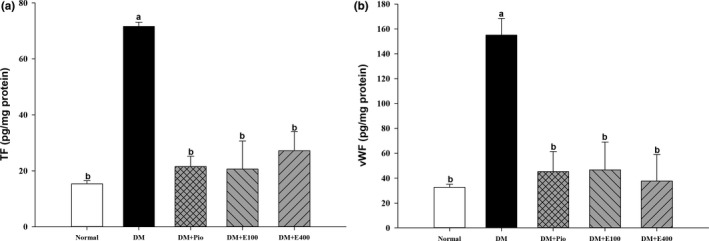
Endothelial tissue factor (TF; a) and von Willebrand factor (vWF; b) in aortas of high‐fat diet and streptozotocin‐induced type 2 diabetic rats treated with *Ruellia tuberosa* L. (RTL) extracts for 4 weeks. Normal: Normal diet; DM: high‐fat diet (HFD; 60% fat) plus STZ (28 mg/kg body weight, i.p.) induced diabetic rats; DM+Pio: DM rats gavaged with pioglitazone (30 mg/kg body weight) for 4 weeks; DM+E100: DM rats gavaged with RTL ethanol extract (100 mg/kg body weight) for 4 weeks; DM+E400: DM rats gavaged with RTL ethanol extract (400 mg/kg body weight) for 4 weeks. Values were calculated as the mean ± *SD*, *n* = 6 for each group. a–b letters = significant differences among all samples tested (*p* < .05)

The TF and vWF levels in the aortic tissues of DM group were higher than those in the normal group. The aorta TF and vWF levels were significantly decreased in the DM+Pio, DM+E100, and DM+E400 groups compared with those in DM group (*p* < .05; Figure 3).

### 3.6 Effect of RTL on SOD and catalase activities in aortic tissues of HFD and STZ‐induced diabetic rats

**Figure 4:**
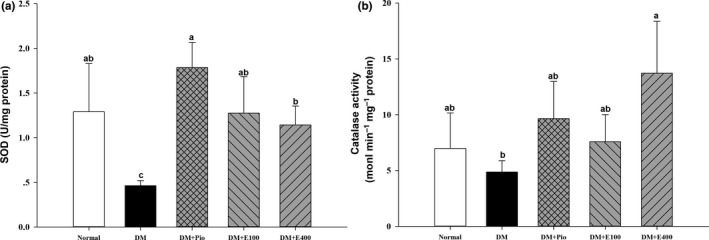
Superoxide dismutase (a) and catalase activity (b) in the aortas of high‐fat diet and streptozotocin‐induced type 2 diabetic rats treated with *Ruellia tuberosa* L. (RTL) extracts for 4 weeks. Normal: Normal diet; DM: high‐fat diet (HFD; 60% fat) plus STZ (28 mg/kg body weight, i.p.) induced diabetic rats; DM+Pio: DM rats gavaged with pioglitazone (30 mg/kg body weight) for 4 weeks; DM+E100: DM rats gavaged with RTL ethanol extract (100 mg/kg body weight) for 4 weeks; DM+E400: DM rats gavaged with RTL ethanol extract (400 mg/kg body weight) for 4 weeks. Values were calculated as the mean ± *SD*, *n* = 6 for each group. a–c letters = significant differences among all samples tested (*p* < .05)

Superoxidase dismutase activity was significantly lower in the aortic tissues of DM group than that in the normal group (*p* < .05). However, the aorta SOD activity in the DM+E100 and DM+E400 groups were higher than in the DM group (*p* < .05) by 174.87% and 146.67%, respectively (Figure 4a). Additionally, catalase activity was also significantly increased in the DM+E400 group compared with the DM group (*p* < .05; Figure 4b).

## 4 DISCUSSION

Excess fat intake or lipid accumulation plays a crucial role in T2DM and obesity. The serum inflammatory markers and FAA levels reportedly increase in T2DM patients over the levels in healthy subjects (Cavelti‐Weder et al., 2012). Excessive FFAs may bind to toll‐like receptors and initiate NF‐κB signaling pathways to generate proinflammatory cytokines. Moreover, FFA also activates c‐Jun amino‐terminal kinase and protein kinase C (PKC), stimulates insulin receptor substrate‐1 (IRS1) phosphorylation, and inhibits phosphatidylinositol‐4,5‐bisphosphate 3‐kinase and Akt activation, resulting in a reduction in glucose transporter type 4 (GLUT4) expression (Kim, 2012), indicating that lipid accumulation is a factor in the creation of IR and hyperglycemia. In the present study, RTL ameliorated dyslipidemia and reduced serum FFA content, indicating that it may further alleviate inflammation in aortic tissues of diabetic rats.

Pio is a hypoglycemic drug belonging to the thiazolidinedione class that promotes fatty acid intake, fat synthesis, and differentiation through the activation of peroxisome proliferator‐activated receptor‐γ (PPARγ) in adipocytes (Kersten, Desvergne, & Wahli, 2000; Vamecq & Latruffe, 1999). Our observations are consistent with the results of a previous study that RTL ethanol extract at dosages of 500 mg/kg for 30 days decreases blood cholesterol, phospholipids, TG, LDL‐C, and VLDL‐C levels while elevating blood HDL‐C concentration in diabetic rats (Ananthakrishnan & Doss, 2013). It has been speculated that RTL may have a similar effect to Pio, which increases the expression of the lipoprotein lipase gene by activating PPARγ expression.

In the present study, it was found that the ethanol extract from RTL increased SOD and catalase activities in the aortas and that such a phenomenon is similar to that in liver tissues of diabetic rats, as observed in our previous study (Chang et al., 2018). High glucose concentration promotes aorta endothelial PKC activation, activates ET‐1 expression, up‐regulates NADPH oxidase, and subsequently enhances ROS production in the smooth muscle cells of diabetic rats. Excess ROS increases intracellular oxidative stress and initiates the aorta endothelial NF‐κB signaling pathway, further leading to inflammatory responses, and subsequently accelerating the progression of atherosclerotic disease (Paneni et al., 2013). ROS also accelerates the synthesis of glucose metabolites, such as methylglyoxal and hexosamine, and affects the dynamic balance of vascular function, which is the major cause of diabetic vascular disease. Hence, a superior antioxidant system that would scavenge excess free radicals is crucial in maintaining vascular tissue health (Paneni et al., 2013). In addition, the activation of PKC also enhances eNOS expression in the aortic tissue of diabetic rats. This result might be ascribed to the production of oxygen ion ($O_{2}^{-}$) and its downstream product hydrogen peroxide (H_2_O_2_) when the vascular tissue is subjected to oxidative stress. Excessive H_2_O_2_ results in increase of eNOS expression (Drummond, Cai, Davis, Ramasamy, & Harrison, 2000). When endothelial cells are injured, NADPH oxidation and eNOS decoupling are increased and $O_{2}^{-}$, peroxynitrite (ONOO^−^), and eNOS coenzyme tetrahydrobiopterin (BH4) of ONOO^−^ become highly sensitive and easily convertible into trihydrobiopterin (BH3) and dihydrobiopterin, leading to eNOS inactivation. BH3 might convert back to BH4 in the presence of an antioxidant, such as vitamin C (Xia et al., 2016). It is thus postulated that the enhancement of aortic antioxidative ability by RTL is involved in lowing ET‐1 and eNOS expression in aortic endothelial cells of diabetic rats.

Furthermore, this study demonstrated that RTL extract reduced aortic inflammation‐related adhesion molecules, including MCP‐1 and VCAM‐1 expression, which revealed their protective benefits (namely reducing foam cell formation from monocytes), and subsequently alleviating vascular endothelial cells damage in the aortas of diabetic rats. Although the NF‐κB‐induced inflammatory response and histopathological examination of the aortic tissue among all groups showed no significant differences (Appendix S1), the RTL extract significantly decreased serum IL‐6 and TNF‐α level, indicating an anti‐inflammatory potential of RTL in diabetic rats. Such anti‐inflammatory properties of the ethanol extract from RTL were also reported in the egg‐induced immune response rodent model (Alam et al., 2009).

Insulin resistance and hyperglycemia in diabetics may increase the formation of blood agglutination and result in vascular thrombosis (Vazzana, Ranalli, Cuccurullo, & Davì, 2012). In this study, RTL extract inhibited the production of aortic TF, a key factor that triggers a coagulation cascade, by decreasing proinflammatory cytokines. The vWF is a large glycoprotein secreted by the endothelial cells. Increase in vWF expression may subsequently cause vascular platelet aggregation, which is the first step in the formation of thrombosis (Frankel et al., 2008). Our observation indicated that RTL extract might ameliorate aorta endothelial damage by reducing inflammatory cytokines and aortic TF, as well as vWF levels, and would subsequently prevent the conversion of prothrombin into thrombin.

High glucose levels promote PKC activation and oxidative stress, and elevate the expression of adhesion molecules and the production of proinflammatory cytokines, thereby causing vascular damage and increased risk of cardiovascular disease (CVD; Ohkita, Tawa, Kitada, & Matsumura, 2012; Paneni et al., 2013). In this study, we observed that RTL extract exhibited the ability to increase antioxidative enzyme activity, to reduce adhesion factors and proinflammatory cytokines contents, and to alleviate endothelial damage‐associated factors levels in the aortic tissues of diabetic rats. Although RTL reportedly contains various phytochemicals, its constituents are quite complicated and have yet to be elucidated. The active components in RTL extracts are currently being isolated and identified in our Taiwan laboratory.

## 5 CONCLUSIONS

**Figure 5:**
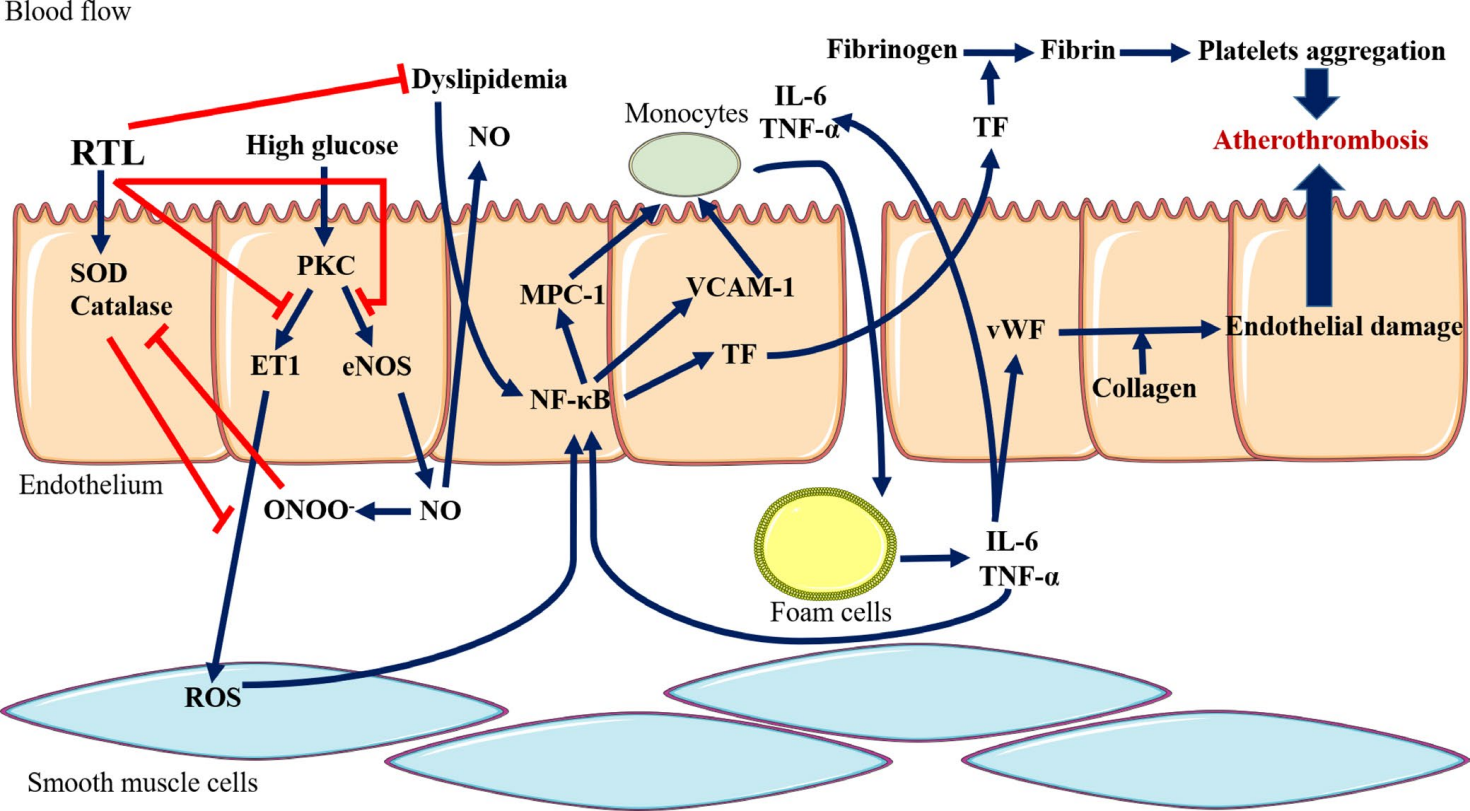
Postulated mechanism of RTL on preventing aorta dysfunction in high‐fat diet and streptozotocin ‐induced type 2 diabetic rats

Vascular endothelial cells damage is attributed to excess oxidative stress and inflammation caused by hyperglycemia. In the present study, ethanol extract from RTL improved dyslipidemia, increased SOD and catalase activity in the aortic tissues of HFD and STZ‐induced T2DM rats. RTL extract also reduced eNOS and ET‐1, and adhesion factors such as MCP‐1 and VCAM‐1, alleviated proinflammatory cytokines such as TNF‐α and IL‐6, and reduced vascular endothelial damage‐associated factors such as TF and vWF in aortic tissues of T2DM rats (Figure 5). These findings suggest that the protective effect of RTL against aorta dysfunction is accomplished by ameliorating dyslipidemia, increasing aortic antioxidative enzyme activity as well as reducing aorta endothelial damage‐associated factors levels, and may possibly alleviate the pathogenesis of other vascular complications in the T2DM paradigm.

## CONFLICT OF INTEREST

The authors declare that they do not have any conflict of interest.

## ETHICAL APPROVAL

The study was conducted in accordance with the ethical guidelines of the Institutional Animal Care and Use Committee of National Taiwan Normal University; Taipei, Taiwan (approval no. 103042).
