# Supplementary material for: Effect of Ruellia tuberosa L. on aorta endothelial damage‐associated factors in high‐fat diet and streptozotocin‐induced type 2 diabetic rats
Source: Food Sci Nutr. 2019 Oct 9;7(11):3742–50. doi: 10.1002/fsn3.1233 (PMC6848814; doi:10.1002/fsn3.1233)
Supplement: Supplementary file 1 [file FSN3-7-3742-s001.doc]

**Supplementary data**

**Histology and immunohistochemistry quantification**

For histopathological examinations, all aortas were fixed in 10% formalin buffer, used routinely paraffin embedding technique. Standard thick sections (3 μm) stained with hematoxylin-eosin (H&E) dye for microscopic observation. There were no significant differences among the Normal group, DM group, DM+Pio group, DM+E100 group, and DM+E400 group (Figure 1S).

Immunohistochemistry (IHC) with antibodies for nuclear factor kappa B (NF-κB) was performed to determine the effect of *Ruellia tuberosa* L. (RTL) on the inflammatory response induced by NF-κB in aortas of high-fat diet plus streptozotocin-induced type 2 diabetic rats. There was no a significant difference between Normal group and DM group. There were no significant differences among the Normal group, DM group, DM+Pio group, DM+E100 group, and DM+E400 group (Figure 2S).

**
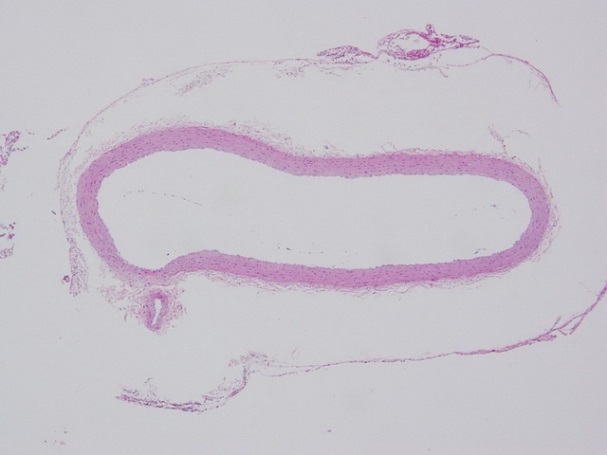

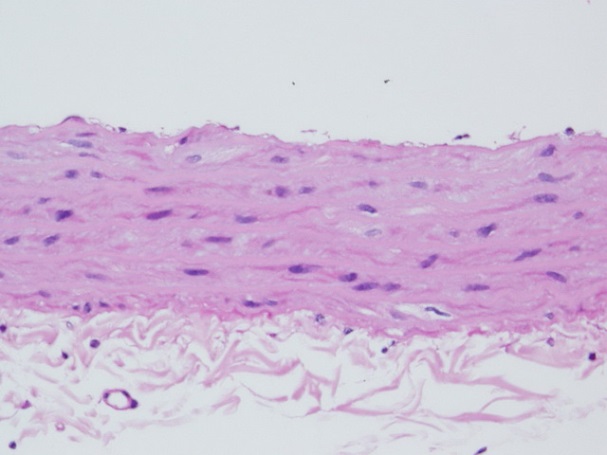
**

Normal (40X)

Normal (400X)

DM (40X)

DM (400X)

DM+Pio (40X)

DM+Pio (400X)

**
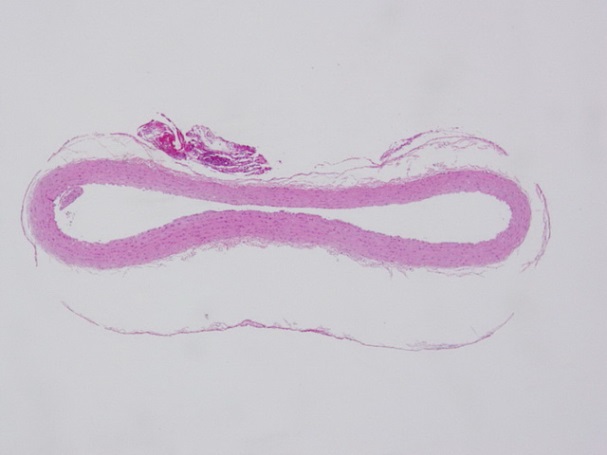

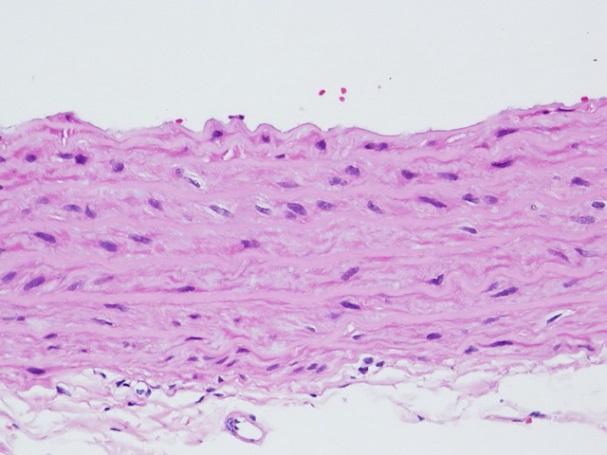
**

**
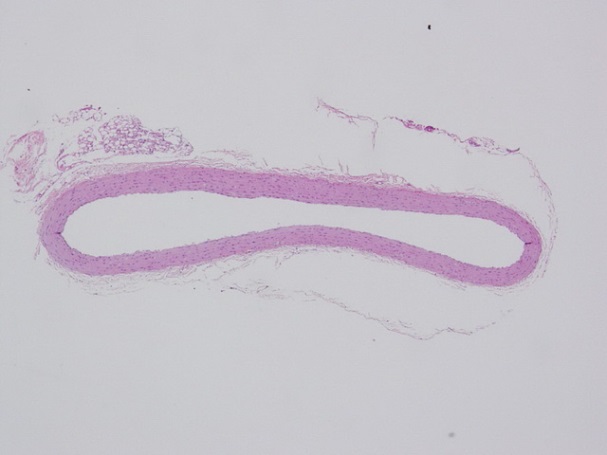

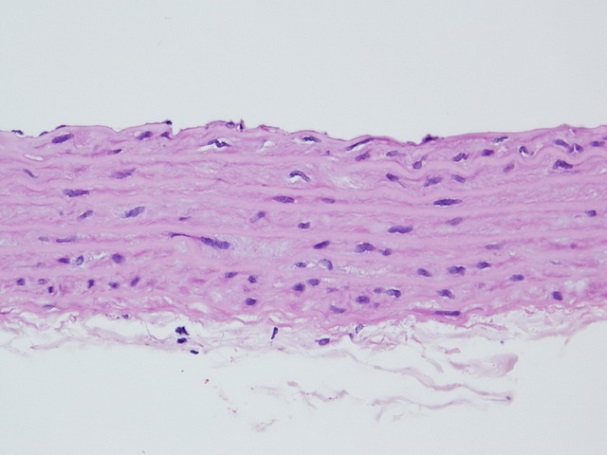
**

DM+E100 (40X)

DM+E400 (40X)

DM+E100 (400X)

DM+E400 (400X)

**
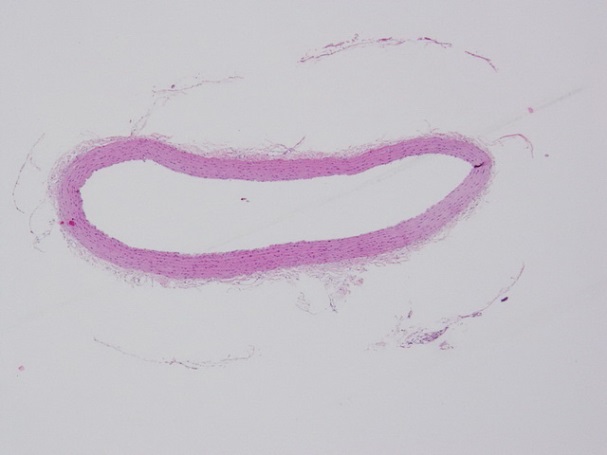

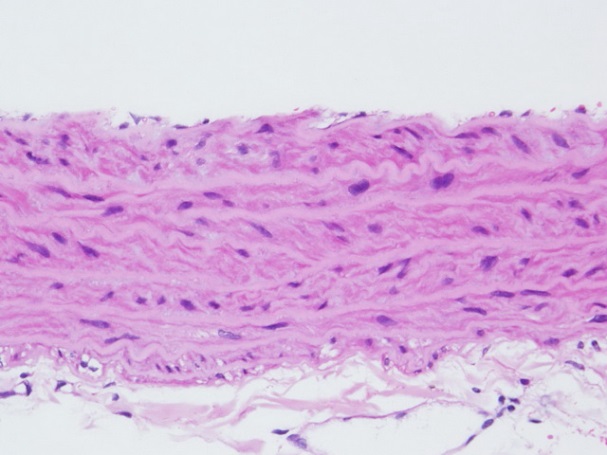
**

**
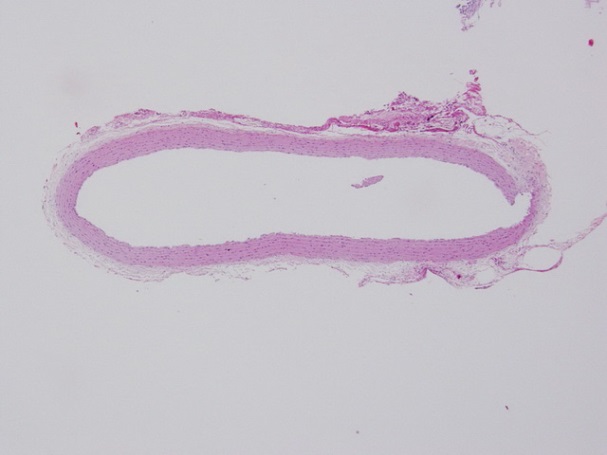

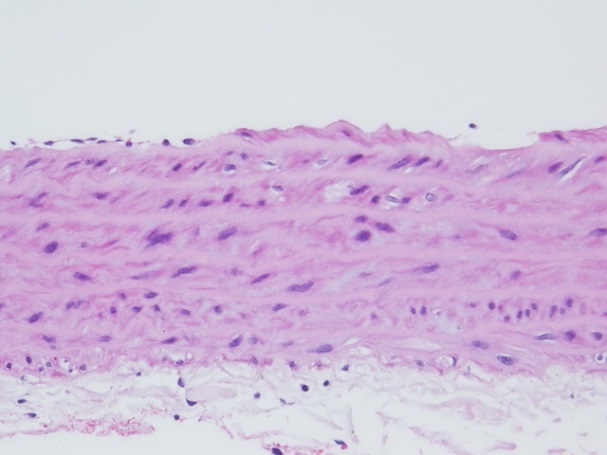
**

**Figure S1. Histopathological analysis of aortas by H&E stain in high-fat diet plus streptozotocin (STZ)-induced type 2 diabetic rats treated with *Ruellia tuberosa* L. (RTL) extracts for 4 weeks.**

Normal: Normal diet; DM: High fat diet (HFD; 60% fat) plus STZ (28mg/kg body weight, i.p.) induced type 2 diabetes mellitus; DM+Pio: DM+Pioglitazone (30mg/kg body weight); DM+E100: DM+RTL ethanol extract (100 mg/kg body weight); DM+E400: DM+RTL ethanol extract (400 mg/kg body weight).

**
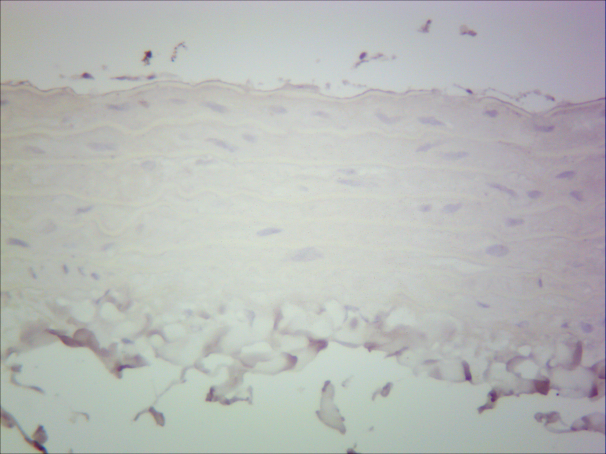

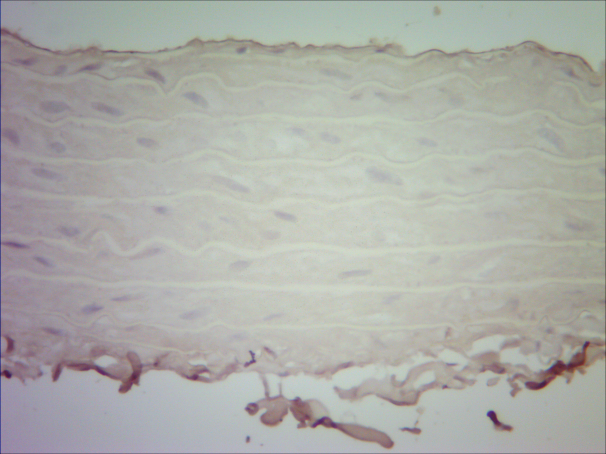
**

**Normal (200X)**

**DM (200X)**

**
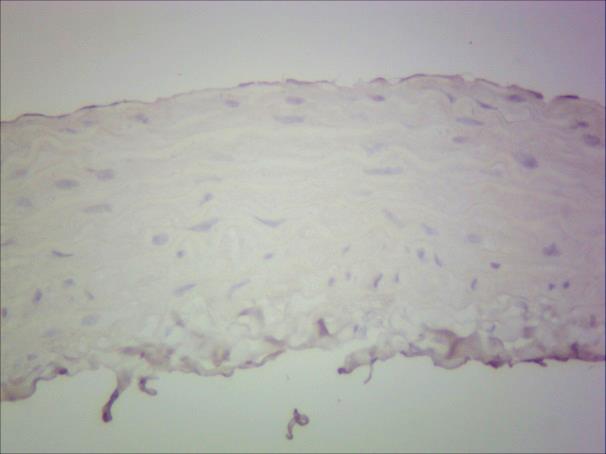
**

**DM+Pio (200X)**

**
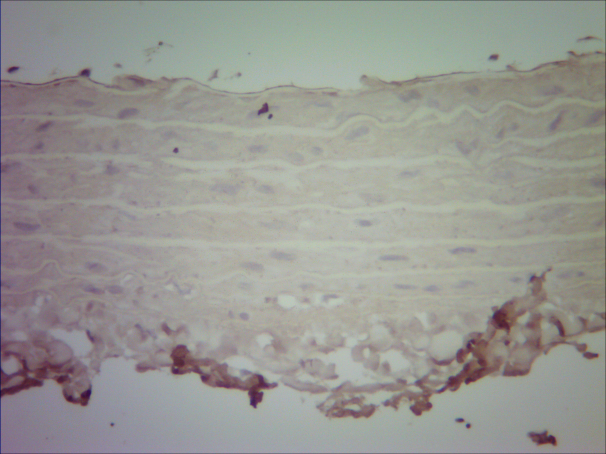

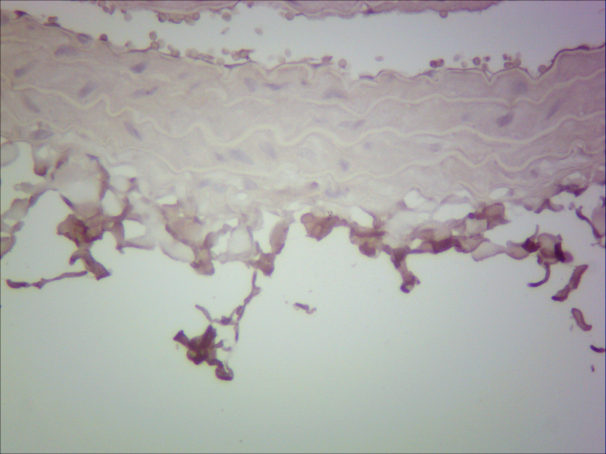
**

**DM+ E100 (200X)**

**DM+ E400 (200X)**

**Figure S2. Immunohistochemistry analysis of aortas by NF-κB stain in high-fat diet plus streptozotocin (STZ)-induced type 2 diabetic rats treated with *Ruellia tuberosa* L. (RTL) extracts for 4 weeks.**

Abbreviations are as in Figure S1.
